# Dispersal limitation determines the ecological processes that regulate the seasonal assembly of bacterial communities in a subtropical river

**DOI:** 10.3389/fmicb.2024.1430073

**Published:** 2024-08-26

**Authors:** Aiping Zhu, Zuobing Liang, Lei Gao, Zhenglan Xie

**Affiliations:** ^1^School of Geography and Tourism, Anhui Normal University, Wuhu, China; ^2^Key Laboratory of Vegetation Restoration and Management of Degraded Ecosystems, Chinese Academy of Sciences, Guangzhou, China; ^3^Lushan Botanical Garden, Chinese Academy of Sciences, Jiujiang, China; ^4^School of Geomatics and Municipal Engineering, Zhejiang University of Water Resources and Electric Power, Hangzhou, China

**Keywords:** bacterial communities, seasonal patterns, ecological network analysis, null model, Liuxi River

## Abstract

Bacteria play a crucial role in pollutant degradation, biogeochemical cycling, and energy flow within river ecosystems. However, the underlying mechanisms governing bacterial community assembly and their response to environmental factors at seasonal scales in subtropical rivers remain poorly understood. In this study, we conducted 16S rRNA gene amplicon sequencing on water samples from the Liuxi River to investigate the composition, assembly processes, and co-occurrence relationships of bacterial communities during the wet season and dry season. The results demonstrated that seasonal differences in hydrochemistry significantly influenced the composition of bacterial communities. A more heterogeneous community structure and increased alpha diversity were observed during the dry season. Water temperature emerged as the primary driver for seasonal changes in bacterial communities. Dispersal limitation predominantly governed community assembly, however, during the dry season, its contribution increased due to decreased immigration rates. Co-occurrence network analysis reveals that mutualism played a prevailing role in shaping bacterial community structure. Compared to the wet season, the network of bacterial communities exhibited higher modularity, competition, and keystone species during the dry season, resulting in a more stable community structure. Although keystone species displayed distinct seasonal variations, *Proteobacteria* and *Actinobacteria* were consistently abundant keystone species maintaining network structure in both seasons. Our findings provide insights into how bacterial communities respond to seasonal environmental changes, uncovering underlying mechanisms governing community assembly in subtropical rivers, which are crucial for the effective management and conservation of riverine ecosystems.

## 1 Introduction

The river ecosystems, which belong to lotic ecosystems, serve as a crucial link between terrestrial and coastal ecosystems, playing a vital role in the material cycle of the biosphere (Ouyang et al., 2020). Bacterial communities, being fundamental and integral components of riverine ecosystems, are widely recognized as primary drivers of biogeochemical cycling, pollutant degradation, and energy exchange (Liu et al., 2021; Yang et al., 2023), thereby assuming key responsibilities in maintaining ecological health and equilibrium of rivers (Kallmeyer et al., 2012; Kuypers et al., 2018). It is well known that inputs of matter and energy from the surrounding environment to aquatic ecosystems exhibit temporal variations that pronouncedly influence bacterial communities (Gao et al., 2017; Xiao et al., 2021). The diversity, structure, and function of bacterial communities are greatly influenced by environmental changes (Sun C. et al., 2021; Sun H. et al., 2021; Wang et al., 2019; Zhang et al., 2021). Seasonal variation has been identified as a representative environmental change factor exerting a substantial impact on bacterial communities in time-series studies (Chen et al., 2023; Liu et al., 2022; Ren et al., 2019), and the response mechanisms of bacterial communities to environmental conditions may exhibit seasonal variability. Consequently, comprehending the seasonal distribution patterns of bacterial communities along a river holds immense significance for deciphering their response mechanisms to environmental changes and enhancing water quality and aquatic ecosystem function.

The processes governing the distribution, diversity, function, and succession of microbial communities are commonly referred to as the community assembly mechanism (Chen et al., 2019; Jiao et al., 2020). The niche-based theory proposes that deterministic processes, including abiotic factors (e.g., environmental filtration) and biotic factors (e.g., interspecific interactions), shape microbial communities (Dini-Andreote et al., 2015; Wang J. et al., 2020). In contrast, the neutral theory assumes that stochastic processes (e.g., birth, death, and dispersion events) govern microbial community assembly (Zhang et al., 2022). Currently, it is universally accepted that both stochastic and deterministic processes contribute to microbial community assembly (Shuwang et al., 2023). Nevertheless, ongoing debates persist regarding the relative significance of these processes in structuring microbial communities (Yi et al., 2021; Zeng et al., 2019). For example, Chen et al. (2019) and Zou et al. (2023) have emphasized stochasticity as the primary driving force behind community assembly, while Wang Y. et al. (2024) have underscored determinism as the main cause. Additionally, it has been reported that the variation of bacterial communities was driven by deterministic processes in winter but dominated by stochastic processes in summer within the same area (Fang et al., 2023). In recent years, a variety of models and algorithms, such as the Sloan neutral community model and null model (Sloan et al., 2010; Stegen et al., 2015; Stegen et al., 2012), have been developed to investigate these assembly processes. These tools provide an opportunity to achieve a comprehensive understanding of the mechanisms driving microbial communities at a process level. Additionally, the noteworthy point is that the majority of microorganisms do not live as separate individuals but form intricate co-occurrence networks through direct or indirect interactions (Ji et al., 2021; Yuan et al., 2022). These interactions have significant impacts on ecological assembly processes (Liu et al., 2023; Wu et al., 2024). Co-occurrence network analysis has proven to be a powerful tool for elucidating species interactions and can offer supplementary insights into microbial community assembly that cannot be obtained through other analytical methods (Fuhrman et al., 2006; Li et al., 2023). In the past few years, there has been considerable focus on investigating the microbial community assembly mechanism and conducting ecological network analysis in diverse lentic ecosystems such as oceans (Yu X. et al., 2023), soils/sediments (Li et al., 2023; Zhao Z. et al., 2022), wetlands (Wang B. et al., 2020), and lakes (Jiao et al., 2020). Compared to these lentic ecosystems, subtropical riverine ecosystems exhibit more pronounced seasonal variations in hydro-chemical conditions and bacterial communities (Sun H. et al., 2021). However, our understanding of how seasonality influences the process governing microbial community assembly in subtropical river ecosystems remains limited. Therefore, studying the seasonal dynamics of co-occurrence networks and ecological processes within bacterial communities in subtropical rivers is a crucial step toward comprehensively understanding riverine microbial ecology and its profound impacts on ecosystem functioning.

The Liuxi River, a major tributary of the Pearl River in the subtropical monsoon region, flows through Guangzhou, one of the fastest-growing regions in the Guangdong-Hongkong-Macao Greater Bay Area (GBA) of China. It holds significant social and ecological importance as it provides water for domestic, agricultural, industrial, and landscape use in Guangzhou. However, rapid urbanization processes witnessed in the past three decades have resulted in severe deterioration in water quality (Xie et al., 2020; Zhu et al., 2020), thereby exacerbating the riverine ecosystem vulnerability and diminishing its ecosystem services value (Ji et al., 2015; Zhou et al., 2018). Moreover, the river exhibits highly dynamic environmental conditions that vary between seasons (Yang et al., 2020). Nevertheless, to the best of our knowledge, the ecological processes shaping bacterial communities and how environmental changes affect microbial community assembly within the Liuxi River have not been understood yet. Environmental variations can influence bacterial communities and their assembly mechanisms by regulating deterministic and stochastic processes (Fang et al., 2023; Obieze et al., 2022). We hypothesize that seasonal variations in environmental conditions (e.g., temperature, pH, dissolved oxygen, organic matter, and nutrients) may exert substantial influence on bacterial communities of the Liuxi River as well as their assembly processes and interspecific interactions. Therefore, the bacterial communities of water samples collected from the Liuxi River were evaluated by 16S rRNA high-throughput sequencing. The objectives of this study are to: (1) investigate seasonal patterns of microbial community structure and diversity, (2) characterize the species interactions and topological properties of bacterial co-occurrence networks, and (3) identify the dominant ecological processes governing microbial community assembly. This study will be essential for understanding the response of bacterial communities to seasonal variations and provide a crucial theoretical foundation for enhancing functionality and promoting the sustainable development of river ecosystems.

## 2 Materials and methods

### 2.1 Site description and sample collection

**Figure 1:**
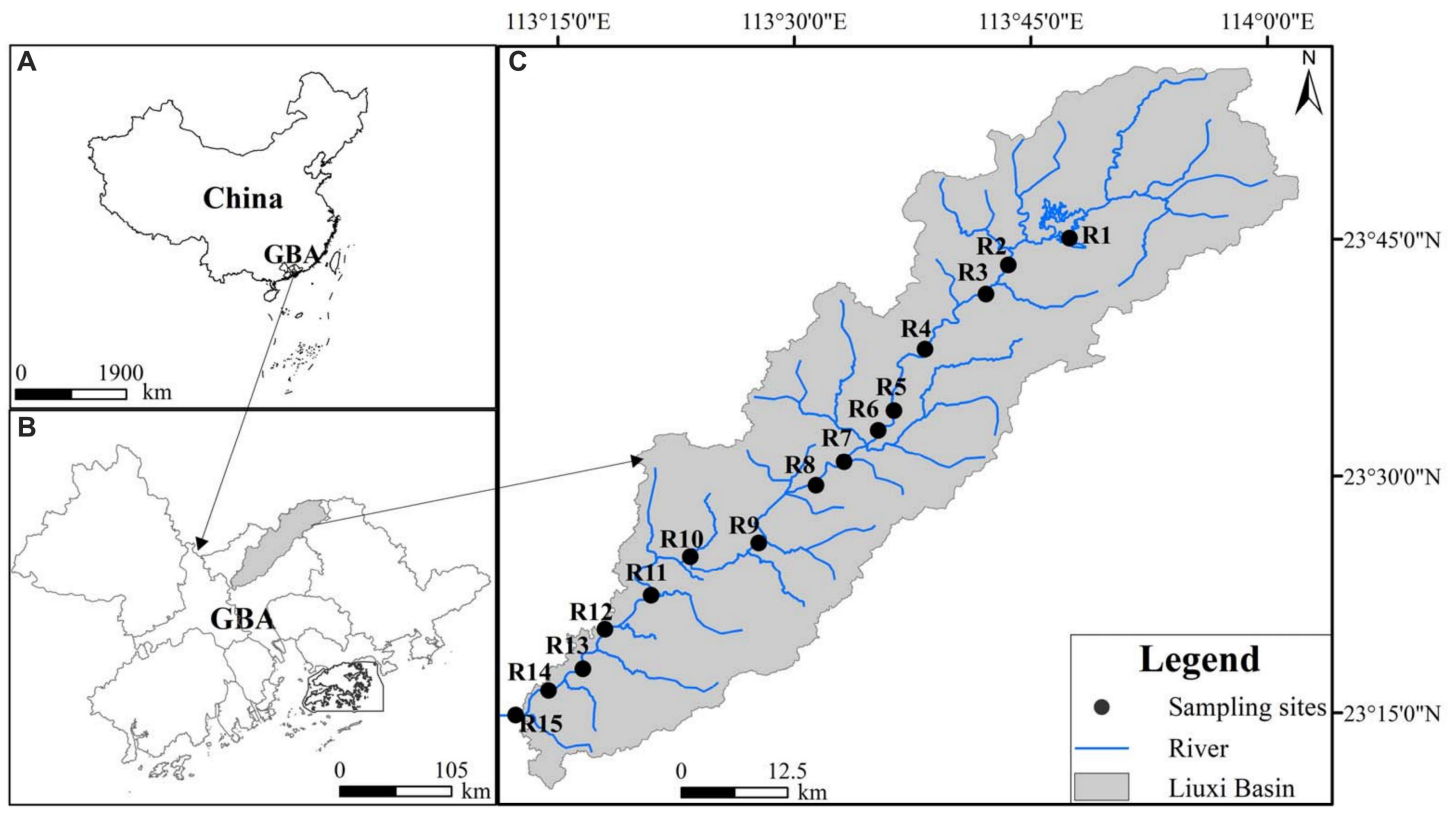
The study area and sampling sites.

The Liuxi River is located in Guangzhou city of GBA (Figure 1), with a length of 171 km and a basin area of 2,300 km^2^ (Zhu et al., 2020). The river basin is located in the subtropical monsoon climate zone, and its average annual temperature and precipitation are 20.3°C and 2,143.8 mm, respectively (Zhu et al., 2020). Due to the effect of the East Asian monsoonal circulation, approximately 80% of the annual precipitation falls during the wet season from April to September (Yang et al., 2020). Since the 1960s, 5 reservoirs and 8 diversion projects have been conducted within this basin, which affects the water levels and hydrological connectivity of the Liuxi River (Yang et al., 2020; Zhu et al., 2020).

### 2.2 Sample collection and analysis

#### 2.2.1 Water sample collection

Thirty surface water samples (R1–R15, Figure 1 and Supplementary Table S1) were collected from the Liuxi River during June 2020 (wet season, WS) and January 2021 (dry season, DS), respectively. Dissolved oxygen (DO), electrical conductivity (EC), oxidation-reduction potential (ORP) and temperature (T) were measured *in situ* using Hach portable meters (HQ40d). The sampling campaigns took place within 2 days for both seasons, with no occurrence of rain events. After collection, the water samples, each with a volume of 1 L, were filtered through 0.22 mm-pore-size polycarbonate filter membranes (Millipore, Billerica, United States). The membranes were then placed into petri dishes and immediately stored at −40°C for DNA extractions. The filtered samples were stored in a refrigerator at 4°C upon arrival at the laboratory. All experimental materials utilized for microbial analysis were sterilized in an autoclave prior to the collection of water samples.

#### 2.2.2 Analysis of environmental factors

The concentrations of ammonium-nitrogen (NH_4_^+^) were determined *in situ* using spectrophotometry (HI96733, HANNA, Italy). Major anions (Cl^−^, SO_4_^2−^, and NO_3_^−^) and the dissolved organic carbon (DOC) were analyzed by ion chromatography (ICS-900, Dionex, United States) and the TOC analyzer (Vario TOC; Elementar, Germany), respectively.

#### 2.2.3 DNA extraction and high-throughput sequencing

The MoBio PowerSoil DNA Isolation Kit (MoBio Laboratories, Carlsbad, CA, United States) was utilized for the extraction of total DNA from water samples following the manufacturer’s guidelines. The concentration of each DNA sample was quantified using a Qubit^®^ 2.0 fluorometer (Invitrogen, United States) and Nanodrop 2000 spectrophotometer (Thermo, Waltham, MA, United States), and quality assessment was conducted through 1% agarose gel electrophoresis (Zhu et al., 2020). Amplification of the V3–V4 region of bacterial 16S rRNA was performed utilizing the universal primer set 515F (5′-GTGCCAGCMGCCGCGGTAA-3′) and 806R (5′-GGACTACHVGGGTWTCTAAT-3′). The PCR mixture (25 μL) contained 10 ng template DNA, 12.5 μL of Phusion High-Fidelity PCR Master Mix with GC buffer (New England Biolabs, Beverly, MA, United States), and 0.5 μL of forward and reverse primers. The PCR reaction conditions were as follows: 94°C for 5 min, followed by 30 cycles of 94°C for 60 s, 57°C for 45 s, 72°C for 60 s, and a final extension at 72°C for 5 min (Jiang et al., 2018). Each sample was amplified in triplicate. Subsequently, PCR amplicons were sequenced on the Illumina MiSeq platform according to standard protocols at Beijing Biomarker Technologies Co., Ltd. (Beijing, China). The raw sequence data were deposited into the National Center for Biotechnology Information (NCBI) database with the accession number PRJNA1131166.

#### 2.2.4 Bioinformatics analysis

The bioinformatics analysis was carried out on the online platform of Biomarker Cloud Platform.^1^ Briefly, the paired-end reads obtained from Miseq sequencing were initially spliced according to their overlap relationships. To obtain high-quality clean reads, the Trimmomatic and UCHIME software were utilized with default settings for raw read analysis to extract effective tags. Subsequently, the UPARSE pipeline was used for taxonomic assignment at the 97% similarity level via the Ribosomal Database Project Naïve Bayesian Classifier v.2.2 trained on the SILVA database (Edgar, 2013; Zhu et al., 2020). Alpha diversity indices (Chao 1 and Shannon) were generated using the QIIME2.^2^

#### 2.2.5 Microbial community assembly analysis

The Sloan neutral community model (NCM) was utilized to predict the occurrence frequency and relative abundance of operational taxonomic units (OTUs), aiming to investigate the potential impact of stochastic processes on bacterial community assembly (Sloan et al., 2010; Zou et al., 2023). Model fitting, including the estimation of the goodness of fit (*R*^2^) and migration rate (*m*), was conducted using R packages “Hmisc,” “minpack.lm,” and “stats4” (Nyirabuhoro et al., 2020; Yu C. et al., 2023). Additionally, a community-level null model analysis was applied to quantify the relative contributions of stochasticity and determinism in microbial community assembly processes (Chen et al., 2022; Stegen et al., 2013). The null model analysis employed two indices: the beta nearest taxon index (βNTI) and the Bray-Curtis-based Raup-Click (RC_bray_). These indices were calculated using “picante” and “ecodist” packages in R software (Stegen et al., 2012). A value of |βNTI| >2 indicates that deterministic processes dominate the community assembly process, encompassing homogeneous selection (βNTI <−2) and heterogeneous selection (βNTI >2). Otherwise, stochastic processes are considered as controlling factors for community assembly. Additionally, when RC_bray_ >0.95, it indicates dispersal limitation, RC_bray_ <−0.95 represents homogenizing dispersal; while −0.95 < RC_bray_ < 0.95 suggests undominated stochastic processes (Liu et al., 2021). To assess potential drivers influencing compositional turnover in microbial communities, we examined correlations between pairwise βNTI values and changes in all measured environmental variables. The statistical significance of these relationships was further tested using a permutation-based partial Mantel test with 1,000 permutations (Gao et al., 2021).

#### 2.2.6 Ecological network analysis

The Molecular Ecological Network Analysis (MENA; http://ieg4.rccc.ou.edu/mena/) based on the random matrix theory (RMT) was performed to evaluate the primary connections among bacterial communities (Deng et al., 2012; Liu et al., 2022; Sha et al., 2023). Co-occurrence networks were constructed using Spearman correlation analysis with a significance threshold of <0.05. To ensure comparability across different seasons, a consistent similarity threshold (*S*_t_ *=* 0.78) was applied during the construction of networks. The MENA pipeline was utilized to calculate the topological properties of these networks, while the Gephi 0.9.2 software was employed for their visualization. The nodes within networks are categorized into four groups based on their values of intra-module connectivity (*Zi*) and inter-module connectivity (*Pi*): peripherals (*Zi* ≤ 2.5, *Pi* ≤ 0.62), connectors (*Zi* ≤ 2.5, *Pi* > 0.62), module hubs (*Zi* > 2.5, *Pi* ≤ 0.62) and network hubs (*Zi* > 2.5, *Pi* > 0.62). Except for peripherals, the other three types of nodes are commonly regarded as keystone species (Deng et al., 2012; Shuwang et al., 2023).

#### 2.2.7 Statistical analysis

The alpha diversity indexes (Chao1 and Shannon) and the beta diversity index (Bray–Curtis) were calculated using the “vegan” package in R software. The differentiation of these indices between seasons was tested using the Kruskal–Wallis method (Yang et al., 2023). Analysis of variance (ANOVA) was employed to assess differences in environmental factors across seasons. Principal coordinate analysis (PCoA) based on the Bray–Curtis distance was performed to investigate the dissimilarities in bacterial community structures between the two seasons (Fang et al., 2023). Similarity analysis (ANOSIM) was conducted to explore the statistical significance of variations in bacterial communities between groups. Bacteria with significantly different abundances in different seasons were identified by STAMP, utilizing a Wilcox rank-sum test with an adjusted false discovery rate (FDR) set at 0.05 (Lv et al., 2022; Sha et al., 2023). The Redundancy analysis (RDA) and Mantel test were applied to examine relationships between environmental factors and bacterial community structure. To eliminate multicollinearity among environmental factors, the variance inflation factor (VIF) was computed and a threshold of 10 was used for VIF to identify redundant variables (Yu C. et al., 2023). These statistical analyses were conducted using R software with packages including “stats,” “vegan,” “rdacca.hp,” and “LinkET” (Lai et al., 2022).

## 3 Results

### 3.1 Physicochemical properties

The physicochemical factors are presented in Supplementary Figure S1. Except for SO_4_^2−^, all other environmental factors exhibited statistically significant differences between the WS and DS (*p* < 0.05). Overall, pH, T, NH_4_^+^, and DOC displayed significantly higher mean values in the WS compared to the DS, while EC, DO, NO_3_^−^, Cl^−^, HCO_3_^−^, and ORP demonstrated opposite trends. Notably, ammonia nitrogen pollution was severe in WS, as NH_4_^+^ concentrations exceeded the class III national surface water quality standard of China (GB 3838-2002) in 100 and 53.5% of the samples during the WS and DS, respectively.

### 3.2 The distribution of diversity, composition and function profiles of bacterial communities

**Figure 2:**
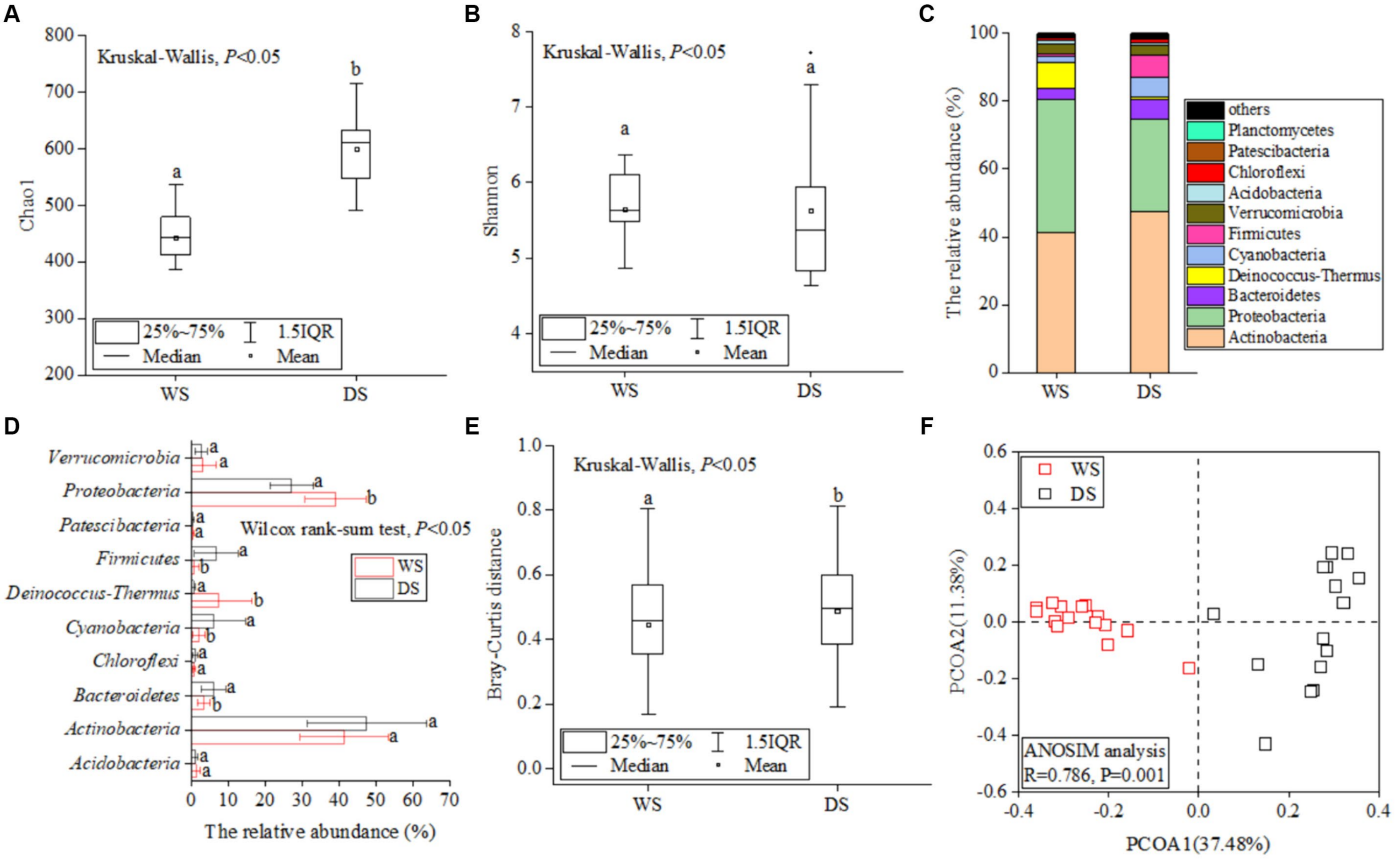
The seasonal distribution patterns of bacterial communities. The differences of Chao1 **(A)** and Shannon **(B)** index between the WS and DS. The relative abundance of bacterial communities at the phylum level **(C)**. Comparative analysis of the dominant bacterial taxa **(D)** across different seasons, significant differences (*p* < 0.05) between the WS and DS are indicated by letters above the bars. The difference in Bray–Curtis distance between the WS and DS **(E)**. Bray Curtis-based PCoA of bacterial community structures between the WS and DS **(F)**.

The global rarefaction curves of water samples (Supplementary Figure S2) collected from the Liuxi River in the WS and DS approached saturation, indicating that the sequencing depth was sufficient for subsequent analyses. After quality control, a total of 1,994,476 high-quality reads were obtained and clustered into 14,060 operational taxonomic units (OTUs) at a similarity level of 97% in 30 water samples. The Chao1 index in the DS (600.6 ± 75.8) was significantly higher than that in the WS (443.2 ± 62.4) (*p* < 0.05, Figure 2A), while the Shannon index also exhibited a higher value in the DS although the difference did not reach statistical significance (Figure 2B). Overall, there was greater alpha-diversity observed within the bacterial community in DS compared to WS.

The OTUs were assigned to 24 phyla, 61 classes, 143 orders, 250 families, and 461 genera. At the phylum level, *Actinobacteria* (44.5 ± 14.3%) was dominant, followed by *Proteobacteria* (33.1 ± 9.3%) and *Bacteroidetes* (4.6 ± 2.9%); these top three phyla accounted for a total of 82.3% of all OTUs (Figure 2C; Supplementary Figure S3). Comparative analysis using STAMP revealed significantly higher relative abundances of *Proteobacteria* and *Deinococcus-Thermus* in the WS compared to the DS (Figure 2D, *p* < 0.05), while *Bacteroidetes* and *Firmicutes* exhibited significantly higher relative abundance in the DS (Figure 2D, *p* < 0.05). Bray-Curtis distances reflecting community similarity were lower in the DS than in the WS (Figure 2E), suggesting a more similar community structure in the WS. Bray Curtis-based PCoA was conducted to display the beta diversity patterns of bacterial communities across different seasons (Figure 2F). The two principal coordinate axes explained >40% of the total variations in bacterial community composition. Samples from the WS and DS were partitioned into two clusters, and the ANOSIM analysis further confirmed significant dissimilarity in the bacterial community structures between the WS and DS (*R* = 0.786, *p* = 0.001).

### 3.3 Relationships between environmental factors and bacterial community structure

The impact of environmental factors on bacterial communities was assessed using the RDA and Mantel test (Supplementary Figures S4A–C). To eliminate collinearity among factors, we calculated the VIF for each environmental factor before analysis. Ultimately, seven factors including pH, T, DO, NO_3_^−^, NH_4_^+^, DOC, and ORP were selected for these analyses (Supplementary Table S2). The RDA reveals significant associations between variations in bacterial community composition (BCC) and pH, T, DO, NO_3_^−^, NH_4_^+^, and DOC. These environmental factors accounted for 53.9% of the variation in BCC, as shown in Supplementary Figure S4A. Importantly, T emerged as the most crucial variable in explaining seasonal variations of BCC, which contributed to the largest variation at 16.6% (Supplementary Figure S4B). The Mantel test demonstrate significant correlations between BCC with pH and T (*p* < 0.01, Supplementary Figure S4C). Furthermore, the Chao1 index exhibited significant negative relationships with pH and T (*p* < 0.001, Supplementary Figure S4C), while showing significant positive correlations with DO (*p* < 0.01, Supplementary Figure S4C), NO_3_^−^ (*p* < 0.05, Supplementary Figure S4C), and ORP (*p* < 0.01, Supplementary Figure S4C).

### 3.4 Ecological assembly processes controlling bacterial communities

**Figure 3:**
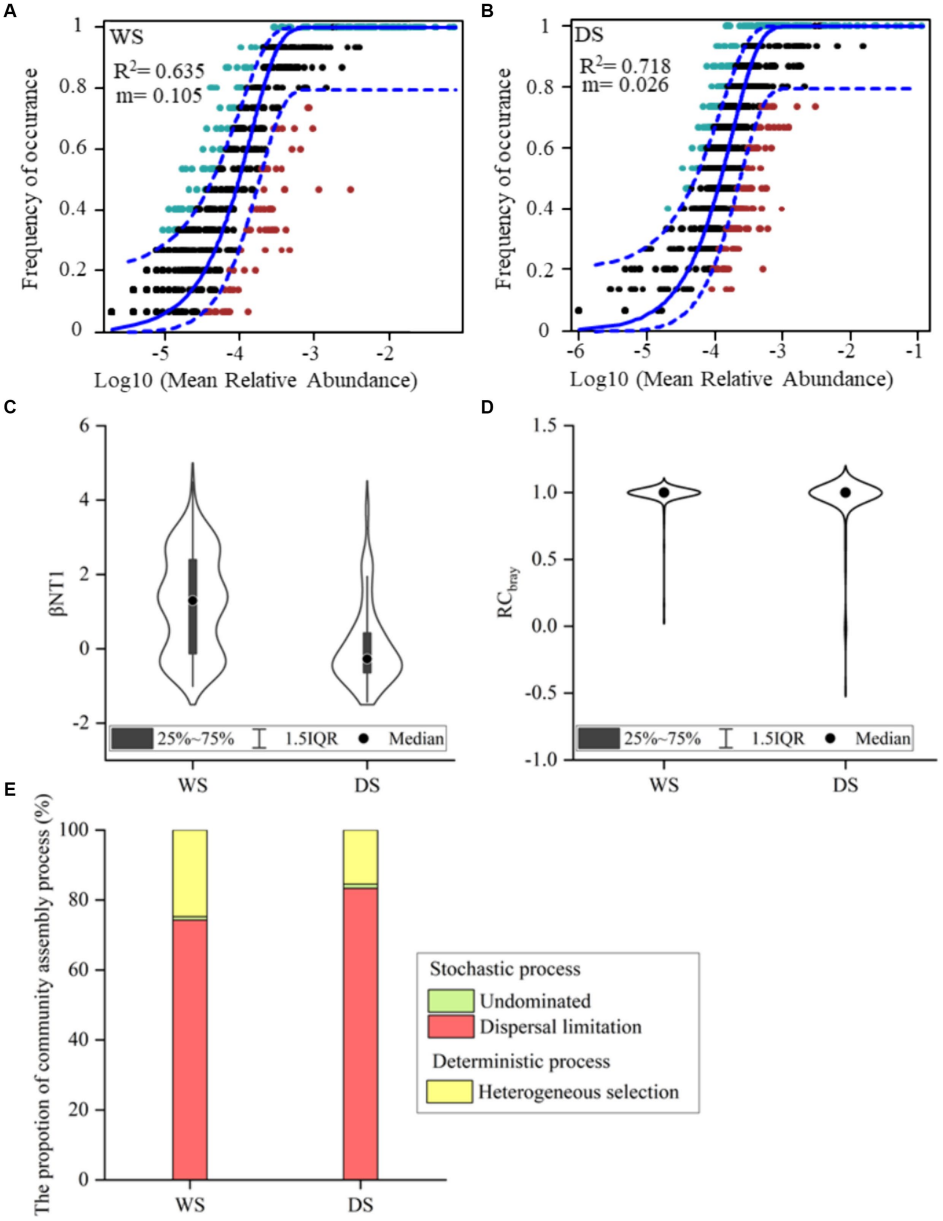
The bacterial community assembly processes. The contribution of stochastic processes to the assembly of bacterial community explained by NCM **(A,B)**. *R*^2^ represents the fit to the neutral model and m indicates the immigration rate of metacommunity. The points falling above and below the 95% confidence interval are colored green and red, respectively, and those within the interval are colored black. The quantification of bacterial community assembly driven by different ecological processes **(C–E)**. βNTI **(C)** and RC bray **(D)** of bacterial communities between different seasons. The percentage contribution of individual processes to community assembly **(E)**.

The NCM successfully estimated a large fraction of associations between the occurrence frequency of OTUs and their relative abundances, explaining 63.5 and 71.8% of community variance in the WS and DS, respectively (Figures 3A,B). These findings suggest that stochastic processes played a crucial role in shaping bacterial community assembly, particularly in the DS. However, since the neutral model failed to explain all variations in the community, deterministic processes also contribute to community assembly. Additionally, there was a noticeably lower migration rate observed in the DS compared to the WS (*m* = 0.026 vs. *m* = 0.105).

The null model analysis was conducted to quantify the relative contributions of stochastic and deterministic processes to community assembly (Figures 3C–E). As illustrated in Figure 3C, the βNTI values of bacterial communities were primarily concentrated within the range of −2 to 2, further confirming that stochastic processes predominantly governed the assembly of bacterial communities in both seasons. However, the relative contributions of stochastic and deterministic processes differed between seasons (Figure 3E). During the WS, stochastic processes accounted for 75.2% of the community variance, with dispersal limitation contributing to 74.3% and undominated processes accounting for only 0.95%. Deterministic processes solely included heterogeneous selection which explained 24.8% of the community variance. During the DS, stochastic processes explained 84.5% of the community variance, with dispersal limitation and undominated processes accounting for 83.3 and 1.21%, respectively, while deterministic processes (solely heterogeneity selection) explained only 15.5% of the community variance.

To gain a more comprehensive understanding of bacterial community assembly, we conducted a linear regression analysis to explore the relationships between changes in environmental factors and βNTI values among samples (Supplementary Figure S5). The findings revealed significant negative correlations between differences in NO_3_^−^ (*R* = −0.377, *p* = 0.000) and DOC (*R* = −0.233, *p* = 0.001) with βNTI. The impact of deterministic processes decreased as levels of NO_3_^−^ and DOC increased. Specifically, the contribution of deterministic processes became negligible when ΔNO_3_^−^ exceeded 1.94 mg/L and ΔDOC surpassed 10.1 mg/L. Notably, ΔNO_3_^−^ had the most pronounced influence on the community assembly.

### 3.5 Co-occurrence network of bacterial communities

**Figure 4:**
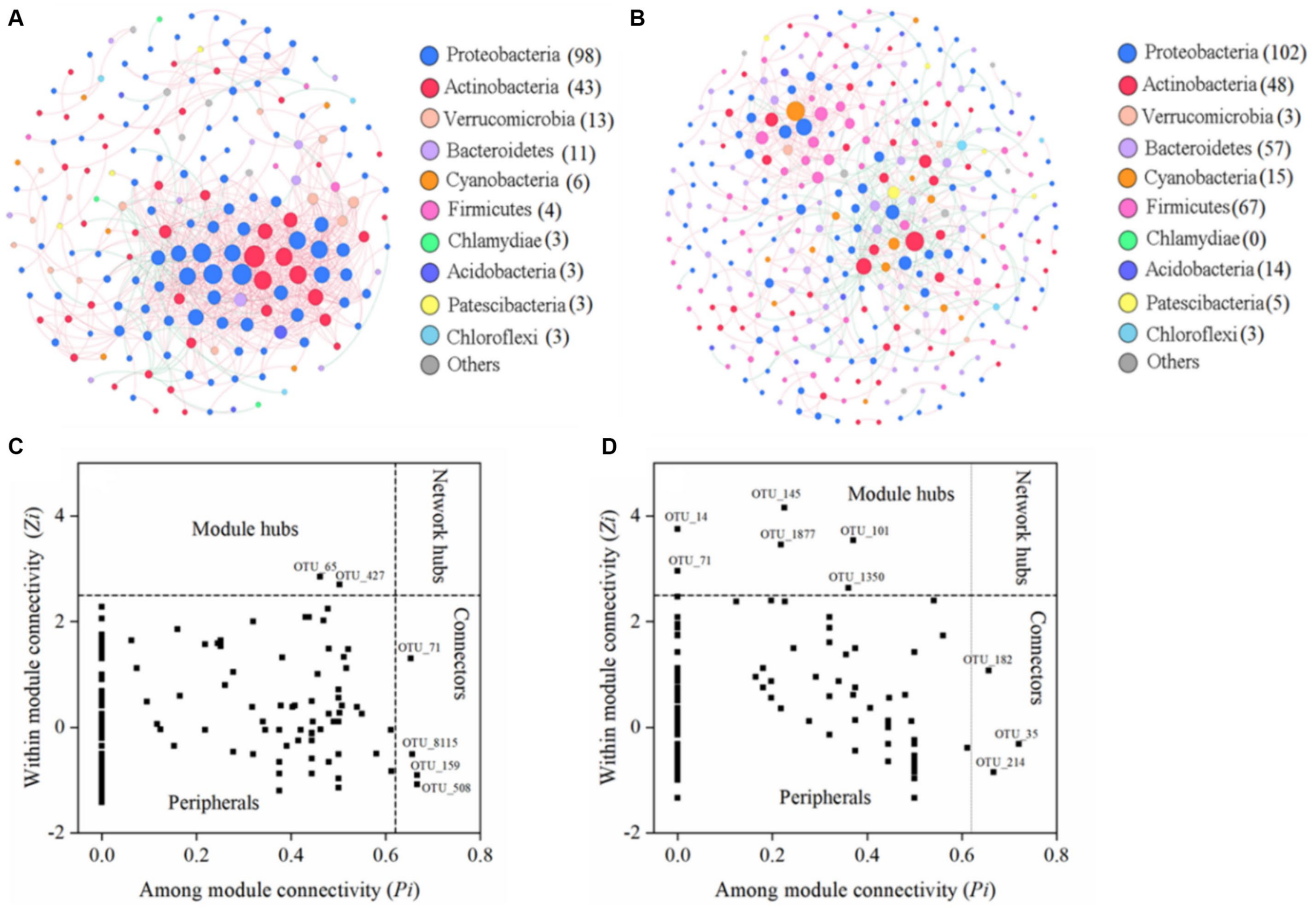
The seasonal co-occurrence patterns of bacterial communities. Network analysis showing the bacterial community interspecies interactions in the WS **(A)** and DS **(B)**. Each node presents one OTU, and the size of node presents the number of connections. Bacteria at the phylum level are colored by different color nodes. The different colors of the edge represent the positive (pink) and negative (green) correlations of species. Identification of keystone taxa in the WS **(C)** and DS **(D)** by their topological characteristics in networks.

The bacterial co-occurrence networks were constructed for the WS and DS (Figures 4A,B), and their topological parameters are presented in Table 1. It was observed that both networks followed a power-law distribution pattern, with *R*^2^ values of 0.795 in the WS and 0.882 in the DS, respectively (Table 1). This indicates that the networks had scale-free and non-random structures. Moreover, both networks demonstrated modular properties as evidenced by modularity values of 0.426 in the WS and 0.690 in the DS (>0.4). Additionally, the networks exhibited predominantly positive correlations among nodes, accounting for approximately 92.9% in the WS network and 64.9% in the DS network.

**Table 1:** Topological properties of bacterial co-occurrence networks in the Liuxi River.

Topological metrics	WS	DS
Nodes	192	321
Edges	813	548
*R*^2^ of power-law	0.795	0.882
Average degree (avgK)	8.47	3.41
Average clustering coefficient (avgCC)	0.303	0.117
Average path distance (GD)	3.75	3.45
Modularity	0.426	0.690
Positive correlation (%)	92.9	64.9
Negative correlation (%)	7.01	35.1

Furthermore, multiple network topological metrics revealed that the co-occurrence patterns differed profoundly between these two seasons (Table 1). In comparison to the DS network, the WS network exhibited a lower GD value (3.75 vs. 5.42), higher average degree (avgK, 8.47 vs. 3.41), and average clustering coefficient (avgCC, 0.303 vs. 0.117), suggesting stronger connections within bacterial communities during the WS. The higher avgCC in the WS further suggests that there were more links within clusters than between clusters. Apart from variations in the network topological indices, distinct differences were observed in terms of network compositions across seasons. The bacterial community network during the WS consisted of 192 nodes and 813 edges, while during the DS, it was comprised of 321 nodes and 548 edges. The nodes in the WS network were primarily composed of *Proteobacteria*, *Actinobacteria*, and *Verrucomicrobia*, while in the DS network, they mainly belonged to *Proteobacteria*, *Firmicutes*, *Actinobacteria*, *Bacteroidetes*, *Cyanobacteria*, and *Acidobacteria*. Additionally, seasonal variations were also observed regarding positive and negative relationships among microorganisms. For instance, *Actinobacteria* was positively correlated with *Proteobacteria* and *Bacteroidetes* in the WS, but negatively correlated with *Proteobacteria* and *Bacteroidetes* in the DS (Figures 4A,B).

The identification of keystone species in the networks was based on the Zi and Pi values (Figures 4C,D). While the majority of nodes (>90%) were classified as peripherals, there were also connectors and module hubs present in both networks that met the criteria for being considered as keystone species. As shown in Supplementary Table S3, the number and composition of keystone taxa exhibited distinct seasonal variations. During the WS, two module hubs and four connectors belonging to *Proteobacteria* and *Actinobacteria* were identified as keystone species, whereas six module hubs and three connectors belonging to *Actinobacteria*, *Cyanobacteria*, *Proteobacteria*, and *Bacteroidetes* were identified during the DS.

## 4 Discussion

### 4.1 Seasonal variations in taxonomic and functional community profile of bacteria

In this study, *Proteobacteria*, *Actinobacteria*, and *Bacteroidetes* were identified as the dominant phyla in the Liuxi River (Figure 2C), which aligns with previous reports of their widespread presence in various riverine habitats (Liu et al., 2021; Zhang et al., 2020; Zou et al., 2023). However, significant differences in relative abundance were observed for *Proteobacteria*, *Deinococcus-Thermus*, *Bacteriodetes*, and *Firmicutes* between seasons (Figure 2). This finding can be attributed to variations in bacterial adaptability to different environment factors such as pH, T, EC, and nutrient concentrations (Xu et al., 2020; Yan et al., 2023). For instance, many members of *Proteobacteria* have been reported as potential nitrifiers and tend to thrive in high ammonia environments (Fan et al., 2020; Wang J. et al., 2024). *Deinococcus-Thermus* belongs to aerobic or facultative anaerobic bacteria primarily found in neutral to alkaline pH with high temperature water bodies (Qing et al., 2023; Sharma et al., 2020). *Bacteriodetes* and *Firmicutes* are commonly enriched in high EC and chloride environments due to their high salt tolerance levels (Miao et al., 2015; Yang et al., 2021; Zhao et al., 2020). The Bray Curtis-based PCoA manifested that bacterial communities from the same season clustered together (Figure 2D), indicating distinct community compositions between the WS and DS. Several reasons may account for this phenomenon. Firstly, the results of RDA and Mantel test indicate that T, pH, DO, NO_3_^−^, NH_4_^+^, and DOC exerted significant effects on bacterial community structures (Supplementary Figure S4). These environmental variables showed substantial variations between the two seasons as depicted in Supplementary Figure S1. Therefore, the pronounced seasonal fluctuations in environmental conditions could be responsible for the differentiation of community structures across seasons. Secondly, in the Liuxi River, rainfall predominantly occurs during the WS, resulting in elevated water levels and increased river flows during this period (Chen et al., 2019; Zhu et al., 2020). The intensified precipitation and river flow during the WS can enhance bacterial diffusion and dilution processes, thereby altering the community composition (Huang et al., 2023b). Thirdly, the seasonal succession of bacterial communities may be driven by species interactions (Chen et al., 2019; Huang et al., 2023b; Yang et al., 2023), as stochastic processes were found to dominate the bacterial community assembly within the river. Moreover, Bray-Curtis distances indicated significantly lower dissimilarity among microbial communities during the WS compared to the DS periods (Figure 2E). This observation can plausibly be explained by the increased hydrological connectivity and dispersal rates facilitated by higher river flow during the WS (Isabwe et al., 2018; Wang et al., 2015); high dispersal rates may contribute to greater homogenization within the microbial community (Pan et al., 2022; Zhang et al., 2022).

Apart from the evident variations in bacterial communities between the two seasons, we also observed the seasonal change in alpha diversity, with a higher level of alpha diversity in the DS, which aligns consistently with previous research findings (Lu L. et al., 2022; Ren et al., 2019; Zhou et al., 2021). The more stable hydraulic characteristics in the DS, compared to the fluctuating aquatic conditions caused by frequent rainfall in the WS, potentially contribute to a favorable environment for microbial reproduction and growth (Lu L. et al., 2022; Zhao et al., 2021). Furthermore, several studies have reported that high concentrations of ammonia nitrogen can inhibit bacteria survival and reduce bacterial diversity (Do et al., 2021; Ning et al., 2023; Wang J. et al., 2024). Therefore, the excessive presence of ammonia nitrogen in the WS may lead to a decrease in microbial diversity. Additionally, during the WS, the mean temperature of river water exceeded 30°C (Supplementary Figure S1), which could exclude species vulnerable to temperature and subsequently decrease microbial diversity (Chen et al., 2022). A previous study has indicated that a neutral pH plays a crucial role in promoting microbial proliferation in aquatic environments (Ji et al., 2022). Therefore, the increase in microbial diversity can also be attributed to the observed neutral pH values during the DS. This notion aligns with our results that the Chao1 index exhibited significantly negative correlations with pH and T (Figure 3C), noting that both pH and T were significantly higher during the WS. However, our findings contradict those of Zeng et al. (2019), who reported higher alpha diversity among bacterial communities during the WS compared to the DS. This discrepancy may arise due to different environmental conditions and hydrological characteristics across different regions (Fang et al., 2023; Wang et al., 2023; Zeng et al., 2019).

### 4.2 Stochastic processes governing the bacterial community assembly

The disentanglement of mechanisms shaping bacterial community assembly is a fundamental prerequisite for elucidating the response of bacterial communities to seasonal environmental changes and has always been a central focus of microbial ecology (Fang et al., 2023; Jiao et al., 2020; Yang et al., 2023). In the study, we employed the NCM and null model to evaluate and quantify the ecological processes. Consistent results obtained from the NCM and null model indicate that stochastic processes predominantly governed bacterial community assembly in both seasons (Figures 3A–E). Our findings were in line with previous studies conducted in river ecosystems. For example, Sun C. et al. (2021) and Sun H. et al. (2021) revealed that distinct stochastic processes dominated the bacterial community structure in the Han River, China. Additionally, Chen et al. (2019) reported that stochastic processes accounted for approximately 85% of variations in microbial communities within the Tingjiang River (China). The null model further revealed that dispersal limitation was the primary ecological control process among stochastic processes in both seasons (Figure 3E). Dispersal limitation refers to the restricted movement and colonization behaviors of individuals in new locations (Qin et al., 2022). It’s well known that microbial communities are dispersed actively or passively by river flow, unable to counteract its unidirectional movement (Chen et al., 2019). The construction of numerous reservoirs and water diversion projects along the Liuxi River since the 1960s has resulted in hydrologically isolated flow paths, which may contribute significantly to the observed substantial dispersal limitation (Dini-Andreote et al., 2015; He et al., 2021). The dominant role of dispersal limitation in controlling microbial assembly has also been documented by Yang et al. (2023). Furthermore, the null model indicated that dispersal limitation played a more influential role in shaping bacterial communities during the DS (Figure 4E). The value of m calculated by the NCM is interpreted as a measure of dispersal limitation, indicating higher levels when bacterial communities are less affected by dispersal limitation. The significantly lower *m* value in the DS further confirms that the impact of dispersal limitation on bacterial communities was more pronounced during this period. This phenomenon can be attributed to variable rates at which microbial communities disperse due to hydrological changes (Milke et al., 2022; Palmer and Ruhi, 2019). During the WS, abundant precipitation and river discharge enhanced the hydrological connectivity, facilitating easier dispersion and long-distance movement of microbes (Lu L. et al., 2022; Sun H. et al., 2021; Yang et al., 2023). Conversely, reduced hydrological connectivity and runoff in the DS hindered the dispersion of bacterial communities (Huang et al., 2023a; Huber et al., 2020).

Furthermore, deterministic processes also partially explained turnover in community compositions, with a higher contribution observed during the WS compared to the DS. It has been well established that environmental heterogeneity greatly influences the relative contribution of deterministic and stochastic processes (Dini-Andreote et al., 2015; Liu et al., 2023; Shuwang et al., 2023; Wu et al., 2024). In our study, the linear regression analysis on changes in environmental factors and βNTI values demonstrates that NO_3_^−^ played a prominent role in driving microbial community assembly, with community determinism decreasing as NO_3_^−^ concentrations increased (Supplementary Figure S5). As an important nitrogen source for microbial growth, NO_3_^−^ directly or indirectly affects metabolisms and growth of microbes (Han et al., 2020; Kutvonen et al., 2015), making it a major factor mediating the balance between stochastic and deterministic assembly processes. Therefore, the observed lower contribution of deterministic processes could be ascribed to the notably higher level of NO_3_^−^ in the DS. Collectively, these findings highlight the linkages of hydrological conditions and environmental variables with community assembly processes that may further influence the diversity, composition, and function stability of bacteria.

### 4.3 Divergent patterns in species interactions between seasons

Species interactions have significant impacts on the structure and diversity of microbial communities (Ji et al., 2021; Lu M. et al., 2022; Zhang et al., 2021). Co-occurrence network analysis facilitates a comprehensive understanding of potential ecological relationships among microorganisms (Du et al., 2023; Huang et al., 2023b). In microbial co-occurrence networks, positive and negative correlations signify cooperative and competitive relationships among microbial species (Li et al., 2023; Yu C. et al., 2023). The network analysis conducted in this study revealed that mutualism or commensalism played a pivotal role in shaping the bacterial community structure, as the proportion of positive correlations exceeded 60% in both networks. From the perspective of the stress gradient hypothesis, cooperative relationships between microbes can act as buffers against environmental stress, thereby enhancing their resilience in harsh environments (Li et al., 2022; Wu et al., 2024; Zelezniak et al., 2015). The Liuxi River was contaminated with ammonia nitrogen, this “harsh” environment likely led to the dominance of cooperative correlations between microbes during both seasons. Synergistic or symbiotic relationships among different microorganisms may have contributed to mitigating the toxicity associated with ammonia nitrogen pollution. It is noteworthy that compared to the DS network, the WS network exhibited a higher proportion of positive correlations due to more severe ammonia pollution, elevated temperature, and unstable hydrological conditions, which made it a more challenging environment for microbes in the WS. Previous studies have suggested that greater microbial diversity promotes species interactions and enhances the connectivity within co-occurrence networks (Fang et al., 2023; Shuwang et al., 2023; Sun H. et al., 2021). However, our findings indicate that although high bacterial community diversity was observed in the DS, species interactions were not as strong as those observed in the WS (Figure 4 and Table 1). One possible explanation is that significantly lower DOC concentrations restricted microbial utilization of carbon resources leading to reduced interactions among microorganisms when faced with limited available carbon resources. The results were consistent with previous studies, which suggested that the availability of environmental resources could have a significant impact on network connectivity (Dai et al., 2022; Huang et al., 2023b; Lu M. et al., 2022). Furthermore, compared to the DS network, the WS network exhibited a lower level of modularity. In microbial ecology, modules can be interpreted as niches, and a higher modularity value indicates a stronger niche differentiation (She et al., 2022; Yan et al., 2023). Therefore, the decreased modularity in the WS suggests that microorganisms share similar niches and interact more frequently. This reduced modularity could be attributed to the lower dissimilarity in the WS, which induces increased microbial co-occurrence patterns and diminished modularity (Widder et al., 2014; Yang et al., 2023). Additionally, the higher modularity observed in the DS implies a more stable and ordered structure of bacterial communities (Ji et al., 2021; Yan et al., 2023). Previous studies have demonstrated that negative associations (competition) within communities can stabilize community co-oscillation and enhance network stability (Coyte et al., 2015; Li et al., 2023; Wagg et al., 2019). Our findings demonstrate that the DS network exhibited a higher proportion of negative correlations than the WS network, providing further evidence for its greater stability. The disparities in bacterial community stability are likely to be associated with the elevated dispersal rates during the WS, as these rates can result in frequent immigration and emigration of microorganisms, disrupting established interaction relationships and consequently reducing ecological network stability (Lu M. et al., 2022; Widder et al., 2014; Yu X. et al., 2023). Moreover, it has been reported that an increased number of keystone species contributes to enhanced stability and orderliness in microbial networks (Jiao et al., 2020; She et al., 2021). Therefore, our identification of more keystone taxa in the DS may also contribute to constructing a more robust network architecture.

Keystone species play essential roles in driving the structure and function of microbial communities (Banerjee et al., 2018; Ji et al., 2021), as their absence can lead to the disintegration of modules or even networks (Wu et al., 2024; Zhao et al., 2016). In our study, we observed that all keystone species in these two ecological networks exhibited remarkably low abundance levels (Supplementary Table S3). This finding aligns with previous studies highlighting the crucial roles of rare taxa in maintaining the stability of microbial interaction networks (Sun C. et al., 2021; Wu et al., 2024; Yan et al., 2023). Notably, there were seasonal shifts in the compositions of keystone taxa in the river. In the WS, *Proteobacteria* and *Actinobacteria* were identified as keystone species, while the DS included *Proteobacteria*, *Actinobacteria*, *Cyanobacteria*, and *Bacteroidetes* as keystone taxa (Supplementary Table S3). *Proteobacteria* and *Actinobacteria* were found to be the most abundant keystone species in both networks. Previous research has emphasized their significant contributions to organic matter degradation and nitrogen cycling processes (Chen et al., 2022; Gao et al., 2021; Ruprecht et al., 2021; Spain et al., 2009). Moreover, these phyla are widely distributed in environments impacted by anthropogenic activities due to their high metabolic diversities and ability to withstand environmental stress effectively (Ohore et al., 2023; Wu et al., 2022; Zhong et al., 2023). Interestingly, *Cyanobacteria* and *Bacteroidetes* were exclusively present as keystone taxa in the DS possibly due to the presence of water hyacinth during this period. It is well-known that *Cyanobacteria* are associated with cyanobacteria blooms in rivers and lakes (Ohore et al., 2023), while *Bacteroidetes* can effectively acquire organics from algae, and their distribution directly correlates with the presence of algal within aquatic environments (Fang et al., 2023; Shen et al., 2020). Additionally, *Cyanobacteria* and *Bacteroidetes* actively participate in biogeochemical carbon and nitrogen cycling processes (Xue et al., 2022; Zhong et al., 2023). Overall, these keystone taxa perform an indispensable role in carbon and nitrogen cycles and hold ecological significance for regulating community structure and maintaining the ecosystem stability of the Liuxi River. Furthermore, *Bacteroidetes* are normally found in human/animal intestines, suggesting that their appearance may be related to domestic wastewater discharge (Wang et al., 2018). Therefore, the exclusive presence of *Bacteroidetes* as keystone taxa in the DS suggests that the river was more susceptible to anthropogenic impacts during this period. Previous studies have reported that water bodies contaminated by anthropogenic activities such as domestic sewage discharge exhibit elevated levels of EC and Cl^−^ (Chen et al., 2014; Zhu et al., 2019). Consistent with this, significantly higher concentrations of EC and Cl^−^ were observed during the DS compared to the WS, further indicating the pronounced impact of human activities on the river. At the genus level, more than half of the identified genera, including *uncultured_bacterium_f_Burkholderiaceae*, *Enterobacter*, *Acinetobacter*, *Actinomadura*, and *uncultured_bacterium_f_Methylophilaceae* are known pathogens associated with human disease (Gao et al., 2017; Zhou et al., 2020). These findings suggest that Liuxi River may provide niches for pathogen growth and dissemination that pose a threat to public health. Therefore, it’s crucial to conduct further investigations into bacterial composition within Liuxi River.

## 5 Conclusion

The composition, co-occurrence patterns, and assembly processes of bacterial communities in the Liuxi River were investigated during the WS and DS. Noticeable differences were observed in the seasonal variation of bacterial community. The relative abundance of *Proteobacteria* and *Deinococcus-Thermus* was significantly higher in the WS, while *Bacteriodetes* and *Firmicutes* exhibited significantly higher relative abundance in the DS. Bacterial communities in the DS showed higher alpha diversity and heterogeneity compared to the WS. The Mantel test and RDA identified pH, T, DO, NO_3_^−^, NH_4_^+^, and DOC as major factors affecting seasonal variation in bacterial communities. Ecological network analysis revealed that bacterial communities in the WS exhibited more interactions and a higher proportion of positive correlations than those in the DS; whereas they exhibited higher modularity and competition in the DS. Furthermore, the number and compositions of keystone taxa also exhibited distinct seasonal variations. Based on *Zi* and *Pi* values, six keystone species belonging to *Proteobacteria* and *Actinobacteria* were identified during the WS, while nine keystone species belonging to *Actinobacteria*, *Cyanobacteria*, *Proteobacteria*, and *Bacteroidetes* were identified during the DS. The results from both the null model and the neutral community model consistently indicate that stochasticity especially dispersal limitation dominated the assembly process for bacterial communities in both seasons; however, during the DS, dispersal limitation exerts a greater influence due to lower immigration rates of bacterial communities. This study extends our understanding of the microbial community ecology in subtropical rivers. However, considering potential future environmental disturbances resulting from climate change and/or human activities on this river ecosystem, periodic investigations on a larger temporal scale are recommended to gain a better understanding of these dynamics.

## Data Availability

The original contributions presented in the study are included in the article/Supplementary material, further inquiries can be directed to the corresponding authors.

## Footnotes

^1^ http://www.biomarker.com.cn/biocloud

^2^ https://qiime2.org/
