# Supplementary material for: Dispersal limitation determines the ecological processes that regulate the seasonal assembly of bacterial communities in a subtropical river
Source: Front Microbiol. 2024 Aug 26;15:1430073. doi: 10.3389/fmicb.2024.1430073 (PMC11381306; doi:10.3389/fmicb.2024.1430073)
Supplement: Supplementary file 1 [file Data_Sheet_1.docx]

**Supplementary Material**

**Dispersal limitation determines the ecological processes that regulate the seasonal assembly of bacterial communities in a subtropical river**

**Aiping Zhu^1, 2, *^, Zuobing Liang^3^, Lei Gao^2,^ *, Zhenglan Xie^4^**

1. School of Geography and Tourism, Anhui Normal University, Wuhu 241002, China

2. Key Laboratory of Vegetation Restoration and Management of Degraded Ecosystems, Chinese Academy of Sciences, Guangzhou 510650, China

3. Lushan Botanical Garden, Chinese Academy of Sciences, Jiujiang 332900, China

4. School of Geomatics and Municipal Engineering, Zhejiang University of Water Resources and Electric Power, Hangzhou, China

*Corresponding author at South China Botanical Garden, Chinese Academy of Sciences, No. 723 Xingke Road, Guangzhou, 510650, P. R. China.

E-mail address: nvtoo@sina.com

**Declarations of interest: none**

Supplementary materials include the following:

Total figures: 5

Total tables: 3

**Fig. S1.** The values of physicochemical factors between the wet season and dry season

**Fig. S2.** The rarefaction curves of water samples between the wet season and dry season

**Fig. S3.** Variations in the composition of bacterial communities at the phylum level between the wet season and dry season

**Fig. S4.** The Relationships between environmental factors and bacterial community structure.

**Fig. S5**. The proportional shifts in ecological processes are indicated by the relationship between βNTI and the difference in each environmental factor by the solid line; the horizontal dashed lines represent the upper and lower significance thresholds at βNTI = 2 and −2, respectively.

**Table S1.** The information of sampling sites.

**Table S2.** VIF values for the environmental factors.

**Table S3.** The details of module hubs and connectors in the wet season and dry season.


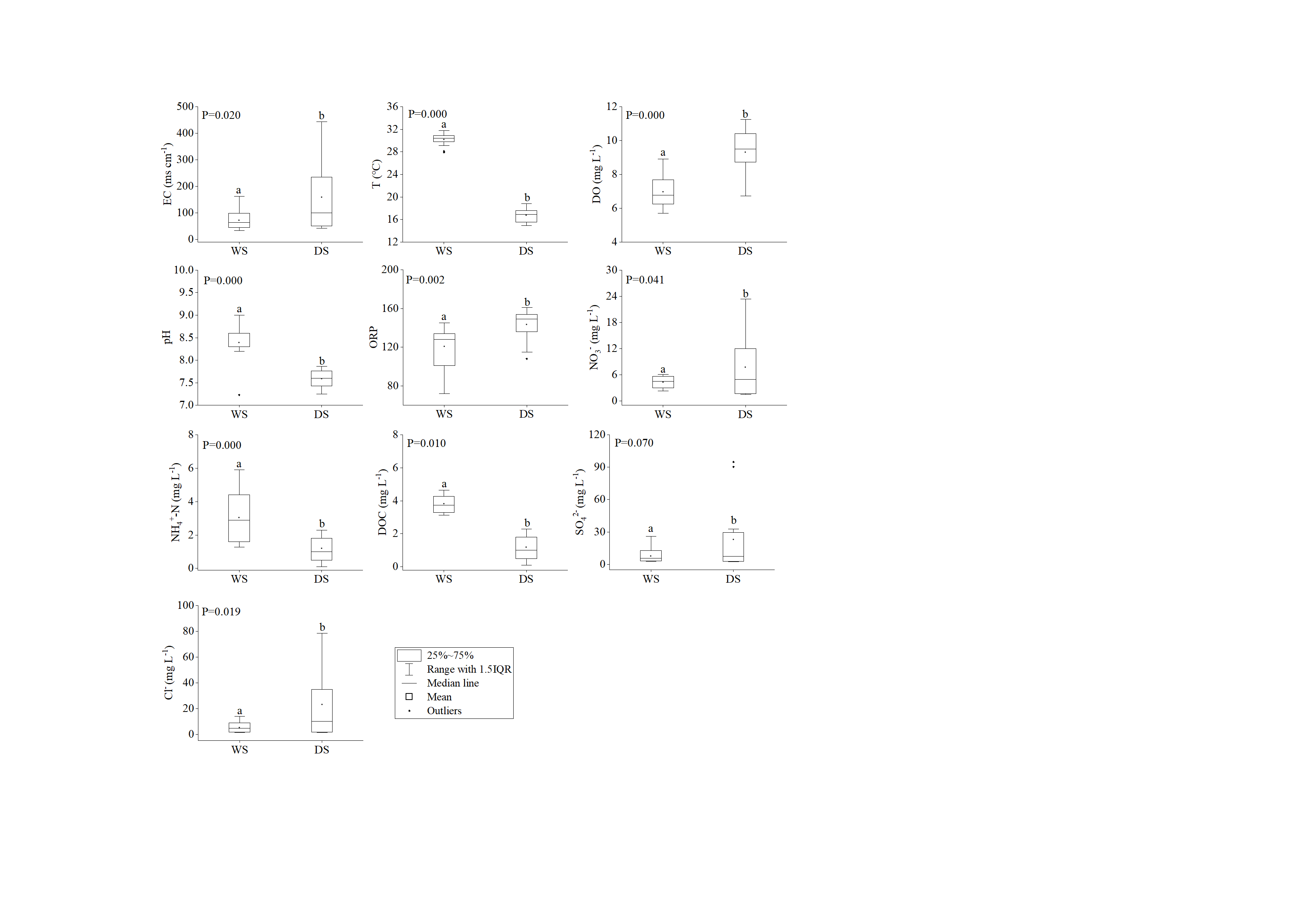


**Fig. S1.** The values of physicochemical factors between the wet season and dry season


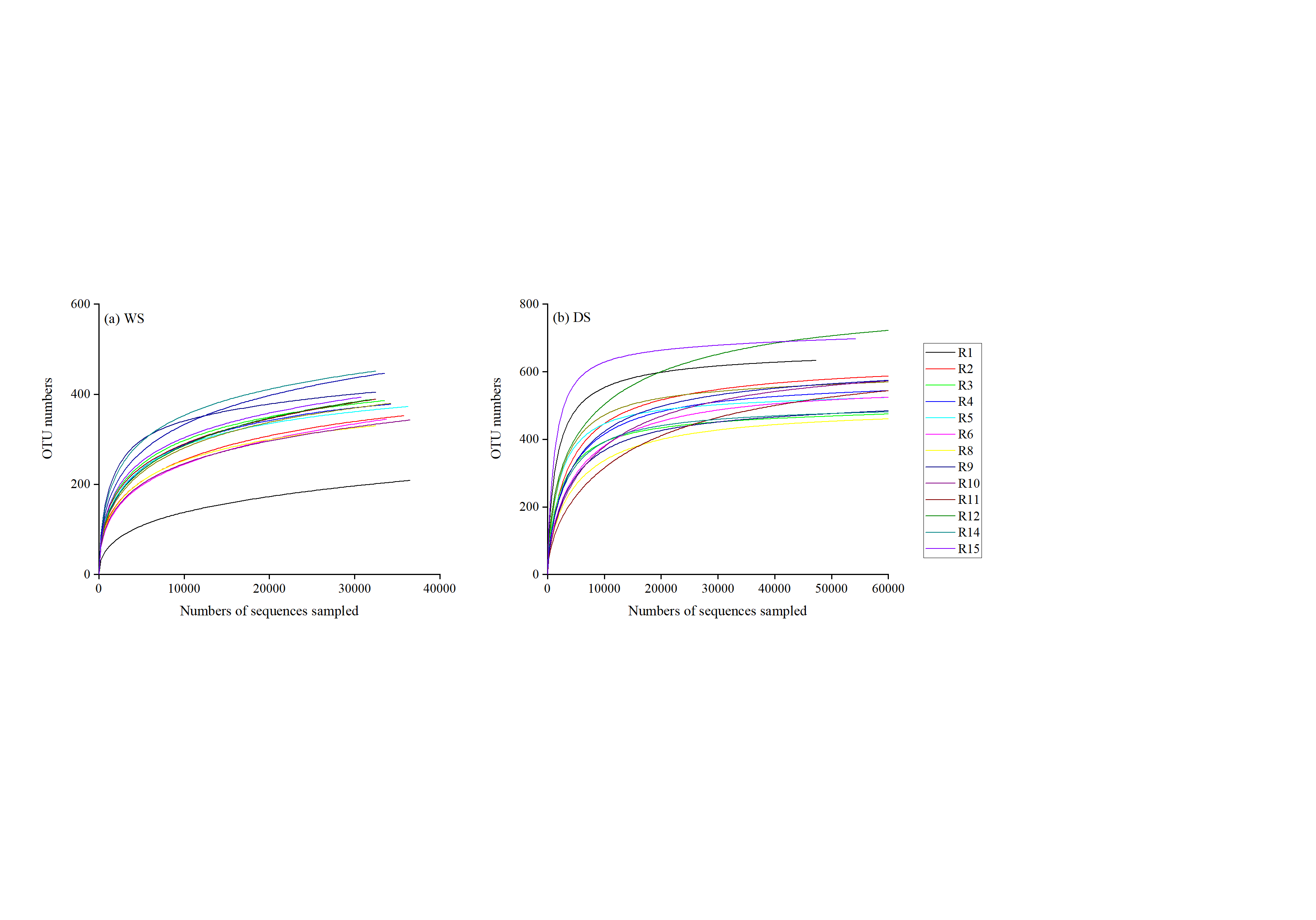


**Fig. S2.** The rarefaction curves of water samples between the wet season and dry season


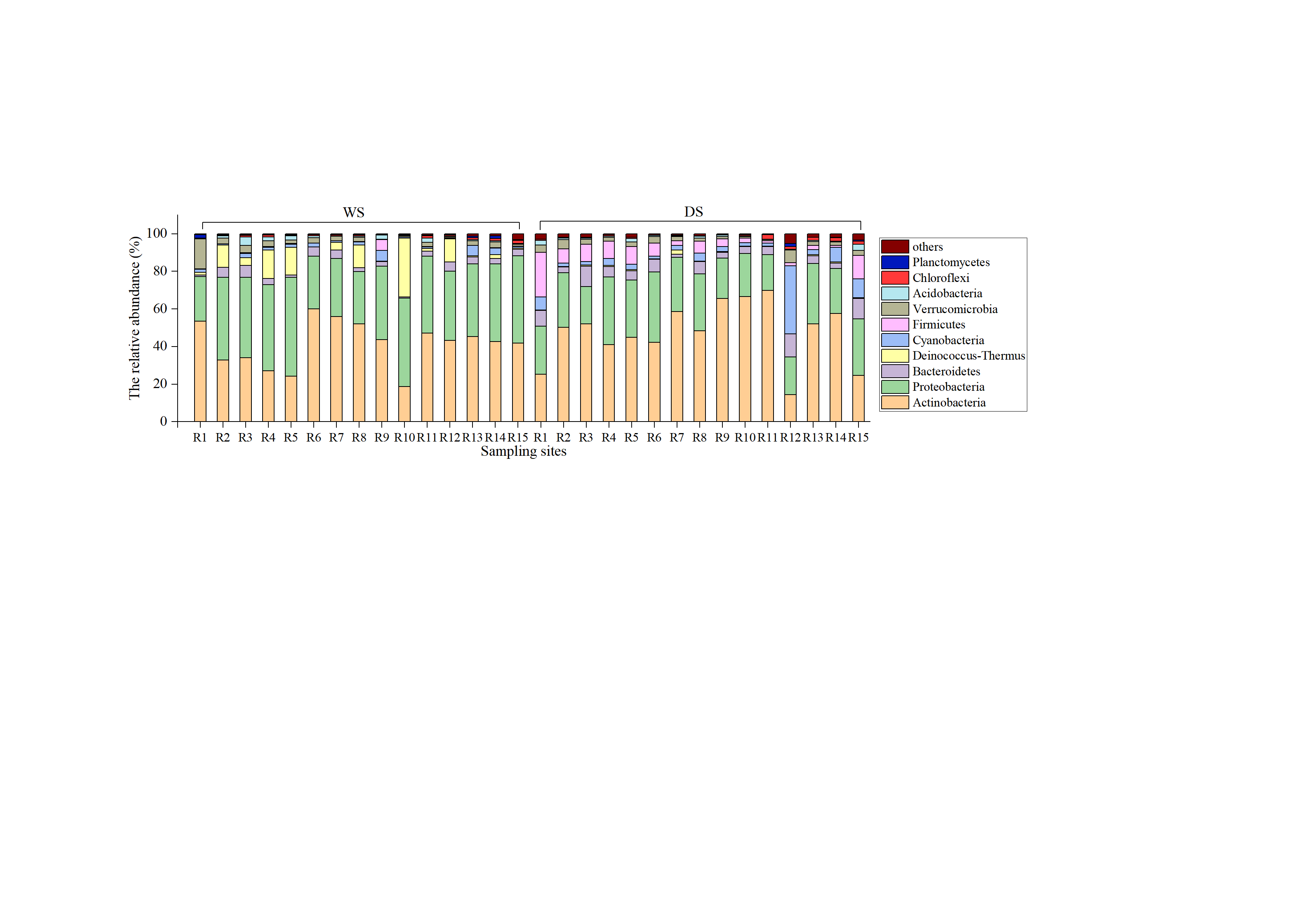


**Fig. S3.** Variations in the composition of bacterial communities at the phylum level between the wet season and dry season.


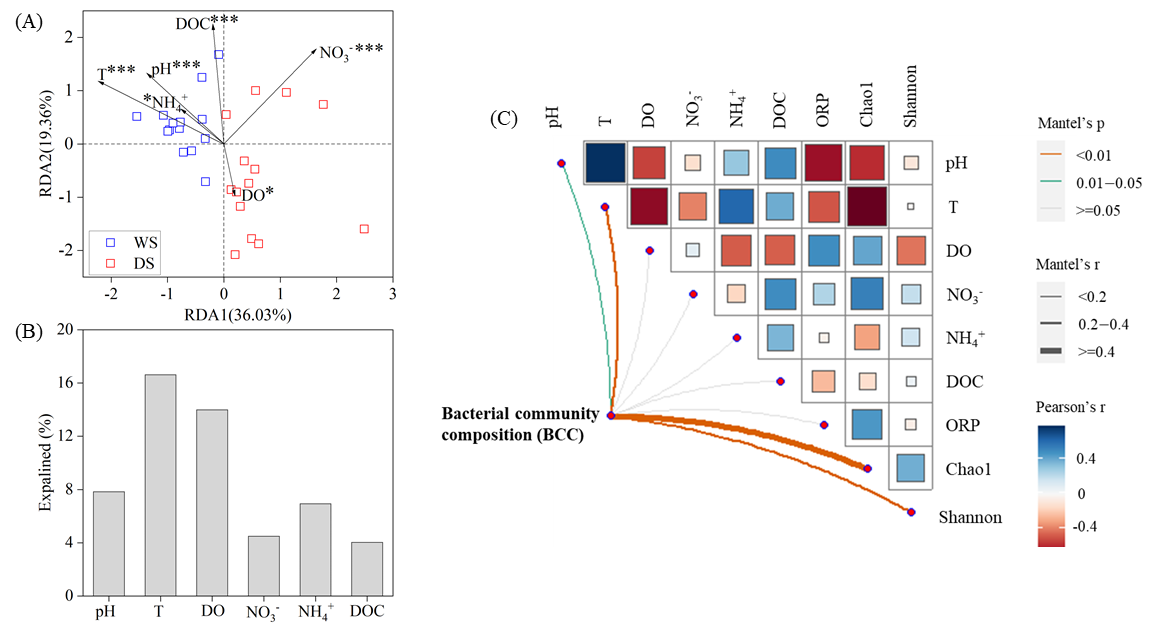


Fig. S4. The Relationships between environmental factors and bacterial community structure. Redundancy analysis (RDA) between bacterial community composition (A) with significant environmental factors. Bar plots are shown below RDA presenting the variation explained by each factor (B). The correlations of bacterial community composition and environmental factors were evaluated using the Mantel test (C).


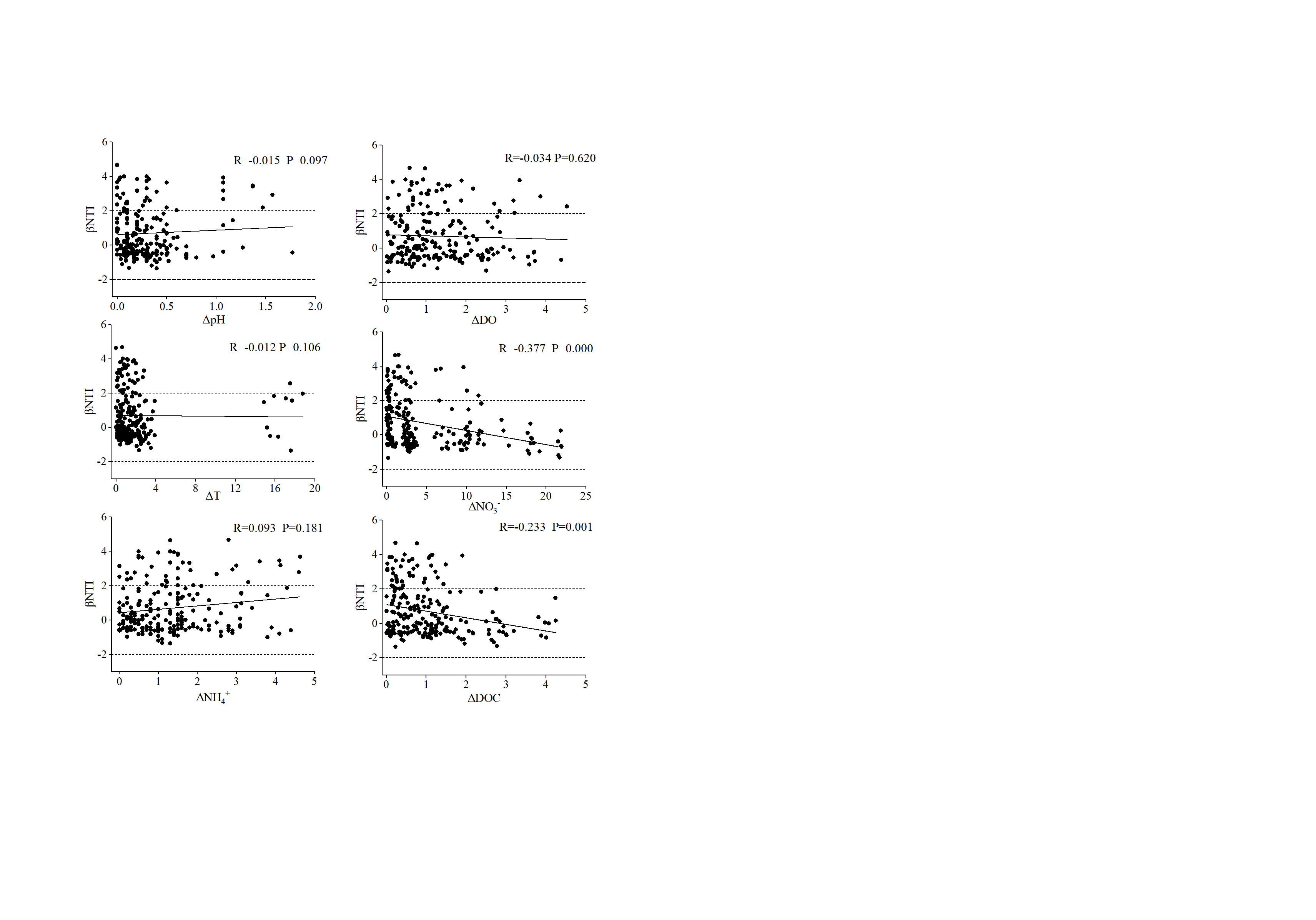


**Fig. S5**. The proportional shifts in ecological processes are indicated by the relationship between βNTI and the difference in each environmental factor by the solid line; the horizontal dashed lines represent the upper and lower significance thresholds at βNTI = 2 and −2, respectively.

**Table S1.** The information of sampling sites

| Name | Longitude (°) | Latitude (°) | Sampling periods |
| --- | --- | --- | --- |
| R1 | 113.800773 | 23.751546 | June 2020, January 2021 |
| R2 | 113.736719 | 23.72514 | June 2020, January 2021 |
| R3 | 113.713341 | 23.694394 | June 2020, January 2021 |
| R4 | 113.651573 | 23.636827 | June 2020, January 2021 |
| R5 | 113.597971 | 23.542743 | June 2020, January 2021 |
| R6 | 113.564626 | 23.523261 | June 2020, January 2021 |
| R7 | 113.534016 | 23.49255 | June 2020, January 2021 |
| R8 | 113.466078 | 23.425145 | June 2020, January 2021 |
| R9 | 113.389686 | 23.414467 | June 2020, January 2021 |
| R10 | 113.354544 | 23.371415 | June 2020, January 2021 |
| R11 | 113.30688 | 23.335168 | June 2020, January 2021 |
| R12 | 113.27526 | 23.297317 | June 2020, January 2021 |
| R13 | 113.246442 | 23.277732 | June 2020, January 2021 |
| R14 | 113.205551 | 23.248134 | June 2020, January 2021 |
| R15 | 113.226859 | 23.157421 | June 2020, January 2021 |

**Table S2.** VIF values for the environmental factors

| pH | T | DO | NO_3_^−^ | NH_4_^+^ | TOC | ORP |
| --- | --- | --- | --- | --- | --- | --- |
| 5.41 | 8.67 | 2.66 | 3.25 | 2.24 | 3.14 | 2.52 |

**Table S3.** The details of module hubs and connectors in the wet season and dry season

| Seasons | Node types | OUT ID | Kingdom | Phylum | Class | Order | Family | Genus | Relative abundance (%) |
| --- | --- | --- | --- | --- | --- | --- | --- | --- | --- |
| WS | Module hubs | OUT_65 | Bacteria | Proteobacteria | Gammaproteobacteria | Betaproteobacteriales | Burkholderiaceae | uncultured_bacterium_f_Burkholderiaceae | 0.957 |
|  |  | OUT_427 | Bacteria | Proteobacteria | Gammaproteobacteria | Betaproteobacteriales | Burkholderiaceae | uncultured_bacterium_f_Burkholderiaceae | 0.165 |
|  | Connectors | OTU_159 | Bacteria | Actinobacteria | Actinobacteria | Micrococcales | Micrococcaceae | uncultured_bacterium_f_Micrococcaceae | 0.007 |
|  |  | OTU_508 | Bacteria | Proteobacteria | Gammaproteobacteria | Xanthomonadales | Xanthomonadaceae | Arenimonas | 0.010 |
|  |  | OTU_8115 | Bacteria | Proteobacteria | Alphaproteobacteria | Sphingomonadales | Sphingomonadaceae | Novosphingobium | 0.099 |
|  |  | OTU_717 | Bacteria | Proteobacteria | Gammaproteobacteria | Enterobacteriales | Enterobacteriaceae | Enterobacter | 0.006 |
| DS | Module hubs | OUT_145 | Bacteria | Actinobacteria | Actinobacteria | Frankiales | Sporichthyaceae | hgcI_clade | 1.242 |
|  |  | OUT_14 | Bacteria | Cyanobacteria | Oxyphotobacteria | Chloroplast | uncultured_bacterium_o_Chloroplast | uncultured_bacterium_o_Chloroplast | 0.789 |
|  |  | OUT_101 | Bacteria | Proteobacteria | Gammaproteobacteria | Pseudomonadale | Moraxellaceae | Acinetobacter | 0.049 |
|  |  | OUT_1877 | Bacteria | Bacteroidetes | Bacteroidia | Bacteroidales | Muribaculaceae | uncultured_bacterium_f_Muribaculaceae | 0.207 |
|  |  | OUT_71 | Bacteria | Proteobacteria | Gammaproteobacteria | Enterobacteriales | Enterobacteriaceae | Enterobacter | 0.103 |
|  |  | OUT_1350 | Bacteria | Actinobacteria | Actinobacteria | Streptosporangiales | Thermomonosporaceae | Actinomadura | 0.002 |
|  | Connectors | OTU_35 | Bacteria | Proteobacteria | Gammaproteobacteria | Betaproteobacteriales | Methylophilaceae | uncultured_bacterium_f_Methylophilaceae | 0.078 |
|  |  | OTU_214 | Bacteria | Bacteroidetes | Bacteroidia | Flavobacteriales | Crocinitomicaceae | Fluviicola | 0.025 |
|  |  | OTU_182 | Bacteria | Proteobacteria | Gammaproteobacteria | Betaproteobacteriales | Burkholderiaceae | uncultured_bacterium_f_Burkholderiaceae | 0.246 |
